# The lipid raft-dwelling protein US9 can be manipulated to target APP compartmentalization, APP processing, and neurodegenerative disease pathogenesis

**DOI:** 10.1038/s41598-017-15128-8

**Published:** 2017-11-08

**Authors:** Renato Brandimarti, Gordon S. Hill, Jonathan D. Geiger, Olimpia Meucci

**Affiliations:** 1 Department of Pharmacology and Physiology, Drexel University College of Medicine, Philadelphia (PA), USA; 2 Department of Pharmacy and Biotechnologies, Alma Mater Studiorum, University of Bologna, Bologna, Italy; 3 Department of Basic Biomedical Sciences, University of North Dakota, School of Medicine and Health Sciences, Grand Forks (ND), USA; 4 Department of Microbiology and Immunology, Drexel University College of Medicine, Philadelphia (PA), USA

## Abstract

The trafficking behavior of the lipid raft-dwelling US9 protein from Herpes Simplex Virus strikingly overlaps with that of the amyloid precursor protein (APP). Both US9 and APP processing machinery rely on their ability to shuttle between endosomes and plasma membranes, as well as on their lateral accumulation in lipid rafts. Therefore, repurposing US9 to track/modify these molecular events represents a valid approach to investigate pathological states including Alzheimer’s disease and HIV-associated neurocognitive disorders where APP misprocessing to amyloid beta formation has been observed. Accordingly, we investigated the cellular localization of US9-driven cargo in neurons and created a US9-driven functional assay based on the exogenous enzymatic activity of Tobacco Etch Virus Protease. Our results demonstrate that US9 can direct and control cleavage of recombinant proteins exposed on the luminal leaflet of transport vesicles. Furthermore, we confirmed that US9 is associated with lipid-rafts and can target functional enzymes to membrane microdomains where pathologic APP-processing is thought to occur. Overall, our results suggest strongly that US9 can serve as a molecular driver that targets functional cargos to the APP machinery and can be used as a tool to study the contribution of lipid rafts to neurodegenerative disease conditions where amyloidogenesis has been implicated.

## Introduction

The clustering of proteins in specific microdomains of plasma membranes on the surface of cells plays a crucial role in regulating receptor activation and internalization, cell function, and behavior. Several lines of evidence point to the contribution of membrane lateral heterogeneity in physiological processes as diverse as immune responses, synaptic activity, cell proliferation, and host-pathogen interactions^1^. Such evidence extended further and demonstrated a key role of lipid rafts - transient microdomains with a lipid and protein composition different than the rest of the membrane^2^ – in these physiological processes. In the brain, lipid rafts can regulate neurocognitive function^3,4^ by modulating key proteins involved in neurotransmission and cell survival such as α-synuclein^5,6^, G-proteins^7,8^, glutamate receptors^9^, and the amyloid precursor protein (APP)^10,11^.

Metabolic processing of APP, a ubiquitously expressed protein, depends on cellular compartmentalization and lipid raft localization^12,13^. During the amyloidogenic processing of APP there is a sequential cleavage of APP by β- and γ-secretases that results in the production of neurotoxic amyloid β (Aβ) peptides^14^. Extracellular accumulation of Aβ generates amyloid plaques in Alzheimer’s disease (AD), whereas alternative cleavage of APP by α-secretase produces neuroprotective fragments^15,16^. Although many additional cellular and metabolic events have been implicated in the neuropathogenesis of AD, the misprocessing of APP and its dependence on lipid-rafts have been consistently reported as signature findings in AD. Interestingly, alterations in the APP pathway have also been identified in other etiologically unrelated pathologies, such as Human Immunodeficiency Virus (HIV)-associated neurocognitive disorders - collectively known as HAND^17–19^.

Although HIV does not infect neurons, it does create a toxic environment for neuronal cells, which alters their survival and function. Indirect effects of viral infection, i.e. inflammation and proinflammatory cytokine release are main contributors to neuronal alterations in HIV patients^20^. Viral proteins have also been shown to directly and negatively affect neurons^21^, and to modify APP processing whereby there is increased accumulation of intraneuronal Aβ^22,23^. Indeed, the HIV envelope protein gp120 can stabilize lipid rafts through enhanced formation of ceramide^24^, an event thought to promote the formation of Aβ. Additionally, gp120 increases the expression and activity levels of BACE1 (beta-site APP-cleaving enzyme: the β-secretase responsible for the initial APP cleavage event in the amyloid cascade) and of APP^25^. The resulting intracellular accumulation of amyloid β peptides in the HIV-infected brain may thus represent a final outcome of increased expression and targeted localization into lipid rafts. However, the causal relation between axonal transport of APP, aberrant Aβ generation, and the related HIV neuropathology has not been fully determined^18,26,27^.

The goal of this study was to generate new molecular tools that can be used to further our understanding of the roles that lipid rafts play in neuronal alterations underlying HAND. To this end, we exploited the properties of the Herpes Simplex Virus 1 (HSV-1) protein US9, which is enriched in lipid rafts, but has no toxic or catalytic activity. US9 is a small type 2 membrane ‘carrier’ protein essential for anterograde spread of viral particles in neurons^28–31^. As we recently demonstrated^32^, the cellular distribution and functional properties of US9 do not require additional viral factors, are solely dependent on US9 sequence, and are not affected by attachment of a reporter/cargo. Furthermore, these US9 behaviors are maintained in multiple cell types, both primary and cultured lines, from different species^32^. In the context of lipid rafts, there are additional features unique to US9 that are important to our long-term goal. First, US9 is likely to be a neutral reporter - unlike another endogenous lipid raft marker, flotillin, which has been shown to interact with BACE1 and affect Aβ processing^33^. Second, it is a transmembrane protein - unlike cholera toxin (CTx), which “indirectly” localizes to lipid rafts through the interaction with the GM1 ganglioside^34^. Though multiple mechanisms may contribute to proper membrane partitioning and cellular distribution of different proteins, a key role of transmembrane domains to membrane partitioning and cellular trafficking has been shown for a number of proteins^35–39^. Notably, while CTx moves in retrograde direction, US9 follows the anterograde path of cellular transport, though it is also recycled from the plasma membrane through the endocytic pathway^40,41^. And third, the physiological function (virus transport) of US9 has been linked to sorting events taking place at the trans-Golgi network. Collectively, US9’s trafficking behavior strongly resembles that of APP in that they both depend on cellular compartmentalization between endosomes and plasma membrane, and on membrane clustering in lipid rafts.

Here, we designed a US9-driven functional assay based on the catalytic activity of an exogenous enzyme, the Tobacco Etch Virus (TEV) Protease, to test the hypothesis that US9 can target APP machinery compartmentalization and metabolism and as a proof of concept for further manipulation of APP processing in the presence of HIV neurotoxins. The results indicate that US9 is able to specifically drive functional cargos to lipid raft domains and is thus a powerful tool for use in characterizing lipid-raft dependent processes that span multiple cellular compartments, such as those that occur in metabolism of APP.

## Results

### US9 efficiently drives recombinant proteins to either leaflet of cellular membranes

**Figure 1:**
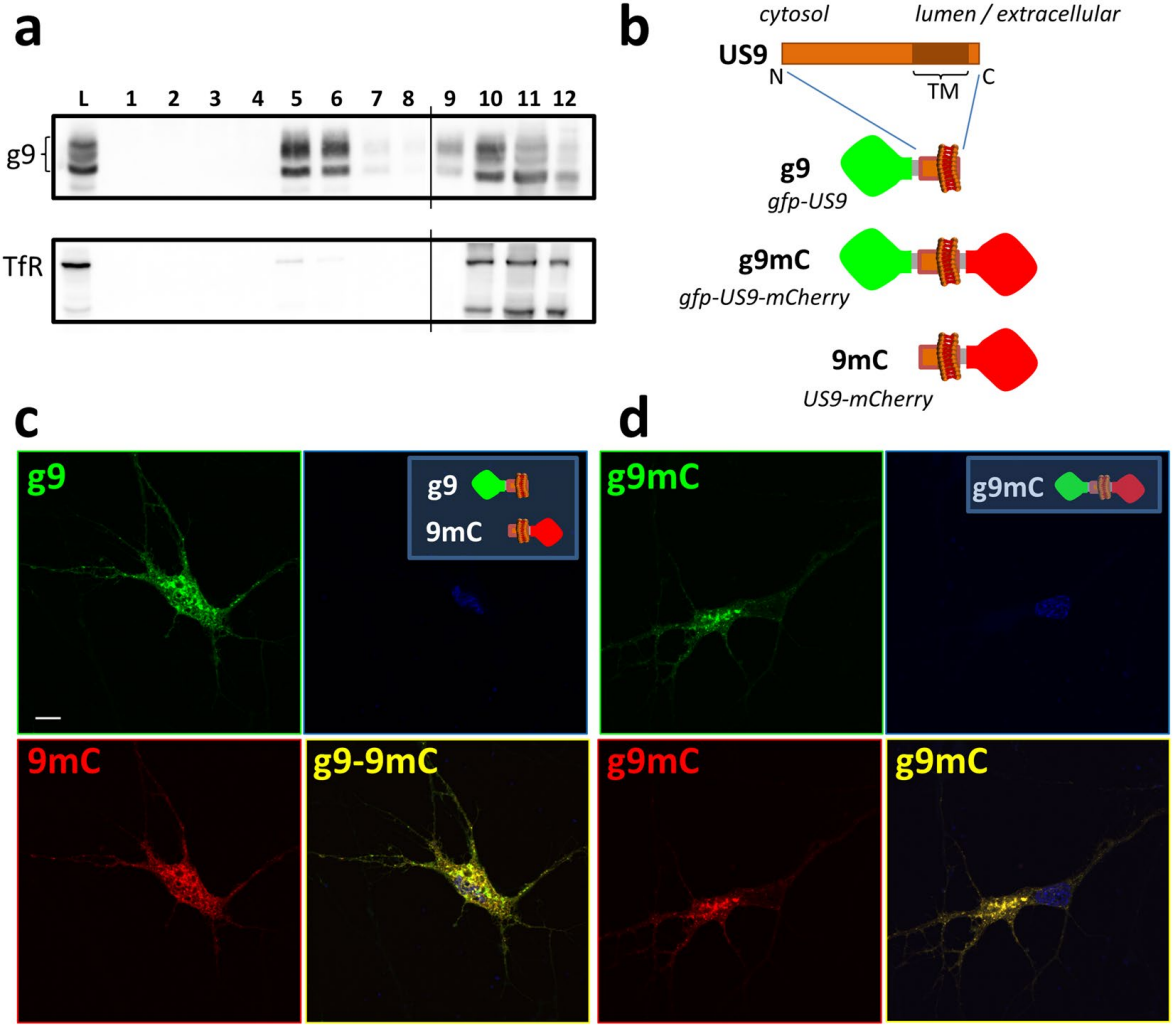
Topological organization and intracellular distribution of US9-driven fluorescent cargos. (**a**) Electrophoretic analysis of proteins from 293T cells expressing gfp-US9 (g9). Cells were solubilized in the presence of cold Triton X100, and separated on Optiprep density gradient. The presence of g9 was detected on each fraction with an antibody against gfp (top gel) and compared to the distribution of the endogenous Transferrin Receptor (TfR) in the bottom gel. Total cell lysates (L) patterns are shown on the first lane, and sequential fractions collected from top are loaded in lanes 1–12. gfp-US9 accumulation in fractions 5–6 indicates its association with lipid rafts. Fractions were loaded on two gels, as indicated by the vertical line between lanes 8 and 9 of each blot. The full-length blots are presented in the Supplementary material (S5). (**b**) Schematic description of the US9 chimeric constructs generated through modifications of the N- and C-terminus. The US9 membrane orientation determines the exposure of gfp and mCherry on the cytosolic and lumenal/extracellular side of the membrane, respectively. (**c,d**) Confocal images of rat neurons showing the intracellular localization of the US9-based fluorescent probes described in (**b**). g9 and 9mC fluorescent patterns were analyzed in (**c**), and their distributions merged in g9-9mC reveal a similar behavior of the two differently oriented constructs. A punctate staining is also obtained in cells expressing the double reporter g9mC (**d**), with a distribution indistinguishable from that of gfp-US9. Scale bar is 10 μm.

Previous studies have shown that modification of the US9 N-terminus by the attachment of green fluorescent protein (gfp) does not alter its function in the context of viral infection^42^. The gfp-US9 fusion protein also retains its properties in the absence of other viral proteins and its natural cargo, the virion^32^. Furthermore, US9 from pseudorabies viruses (PRV) was found to associate with lipid rafts^43^. To confirm that this property is maintained by HSV gfp-US9, we analyzed its behavior on a sucrose gradient (Fig. 1a), a well-established test assessing the ability of proteins to co-fractionate with lipid rafts^44–46^. Membrane proteins that are not solubilized, so called detergent resistant membranes, in the presence of cold non-ionic detergent such as Triton X-100 will float upward in a sucrose gradient and accumulate in fractions that are separated from those containing detergent sensitive membranes. Results from this experiment showed, as expected, that gfp-US9 (i.e. g9) was highly enriched in fractions that corresponded to lipid rafts. In the same samples, the transferrin receptor, a transmembrane protein widely used as a non-lipid raft marker^47^, was mostly found in detergent sensitive non-lipid raft fractions.

Our next goal was to establish whether we could use the non-cytosolic C-terminus of US9 to specifically target functions occurring on the luminal side of vesicles (or on the extra-cellular side of the plasma membrane after vesicles fusion to the plasma membrane). We generated new constructs with the fluorescent reporter protein mCherry attached to the US9’s C-terminus (named 9mC). We also fused the mCherry sequence to gfp-US9, to yield a double reporter of US9 distribution that harbors two different fluorescent probes – i.e. on the N and C terminals of US9 (g9mC). The topological organization of these constructs with respect to membrane insertion is shown in Fig. 1b. The US9-mCherry construct (9mC) was expressed in primary neurons together with gfp-US9 (g9), and results of this experiment are reported in Fig. 1c. The punctate staining of US9-mCherry indicates a vesicular localization of the protein, scattered throughout the cytosol, with an increased accumulation in a region resembling the Trans-Golgi Network. This general distribution implied that mCherry fusion to US9 C-terminus did not alter US9-membrane insertion neither caused aggregation. More importantly, US9-mCherry signal overlapped closely with that of gfp-US9, indicating that N- or C- terminal modifications of US9 are both compatible with US9 targeting capabilities.

The ability of US9 to act as a transport protein independently of the side on which the exogenous sequence is attached is further reinforced by the results obtained with the gfp-US9-mCherry chimera (Fig. 1d). Although we do not have a US9 marker to which we can directly compare the fluorescent profile of g9mC, the analysis of its distribution strongly resembles that of gfp-US9. Additionally, no fusion protein aggregation or cell damage was detected, suggesting that the addition of the two fluorescent cargos does not interfere with the physiological properties of US9.

**Figure 2:**
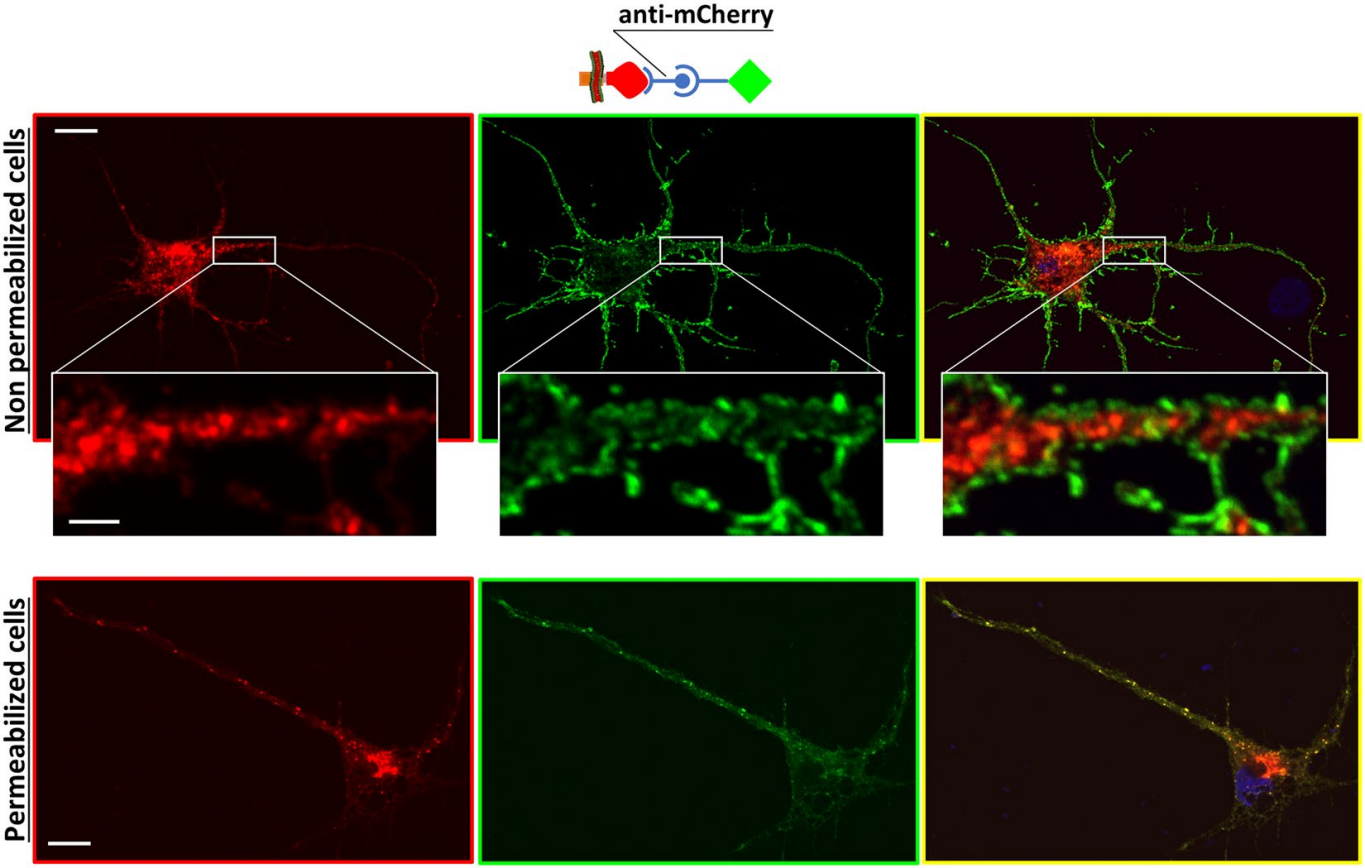
Membrane orientation of US9 C-terminal cargos. Rat neurons expressing 9mC were incubated with the anti-mCherry antibody, in the absence and in the presence of permeabilizing agents. The presence of mCherry on the non-cytosolic side of the membrane was revealed using a fluorescent secondary Ab, as represented in the schematic cartoon. The plasma membrane signal (in green) shown in the central panels indicates the correct orientation of the fluorescent molecule attached to the C-terminus of US9. The mCherry portion of 9mC (left panels, in red) is not accessible to the antibody in non permeabilized cells, and no overlap between mCherry and anti-mCherry from 9mC in intracellular vesicles can be seen in the merged images on right panels. Insets show higher magnifications of the boxed region of the same cell. In permeabilized cells in bottom images, intracellular distribution of 9mC (revealed by the red fluorescence on the left micrograph**)** overlaps the immunodetection obtained with the antibody against mCherry (central image, in green; merged image on the right), at both the plasma membrane and intracellular vesicles. Scale bar is 10 μm (2 μm in insets).

The correct orientation of the newly generated US9-mCherry was additionally tested using immunofluorescence with an antibody directed against mCherry. In this orientation, mCherry is present on the non-cytosolic side of membranes, i.e. vesicle lumen and extracellular space. Therefore, if cells are not permeabilized, the plasma membrane should be labeled with fluorescent antibody while mCherry on vesicles should not be accessible. Our results (Fig. 2, top panels) confirmed the correct orientation of US9-mCherry expressed in rat neurons, with a typical plasma membrane staining with the mCherry antibody, and complete absence of immunofluorescent signal from mCherry-populated intracellular vesicles. The same vesicles became accessible to the antibody when cells were permeabilized (Fig. 2, bottom panels), with overlapping distributions of mCherry (red) and anti-mCherry (green) immunodetection. The cytosolic exposure of gfp in gfp-US9 was confirmed by the experiment in which the chimeric protein was detected with an antibody against the hemagglutinin (HA) tag inserted between gfp and US9. In the absence of membrane permeabilization, HA was not available and no signal was detected (Figure S1, center panels). These results indicate that attachment of an exogenous sequence to either side of US9 does not alter its distribution in transfected cells. Consequently, US9 is able to target exogenous proteins to both leaflets of cellular membranes.

### Design, construction and analysis of a US9-driven functional assay

The US9 autonomous targeting properties described so far using confocal microscopy and biochemical means suggest that US9 is well-suited for studies intended to analyze the intracellular trafficking and distribution of lipid-raft targeted proteins, such as those involved in APP processing. However, in order to demonstrate that US9 can localize in close proximity of these proteins (i.e. sufficiently to interact with them as would be necessary for future interventional experiments using US9-driven enzymes), we developed a US9-based functional assay. In so doing, we also establish the US9’s ability to target a cargo that retains functional activity to specific cellular districts.

**Figure 3:**
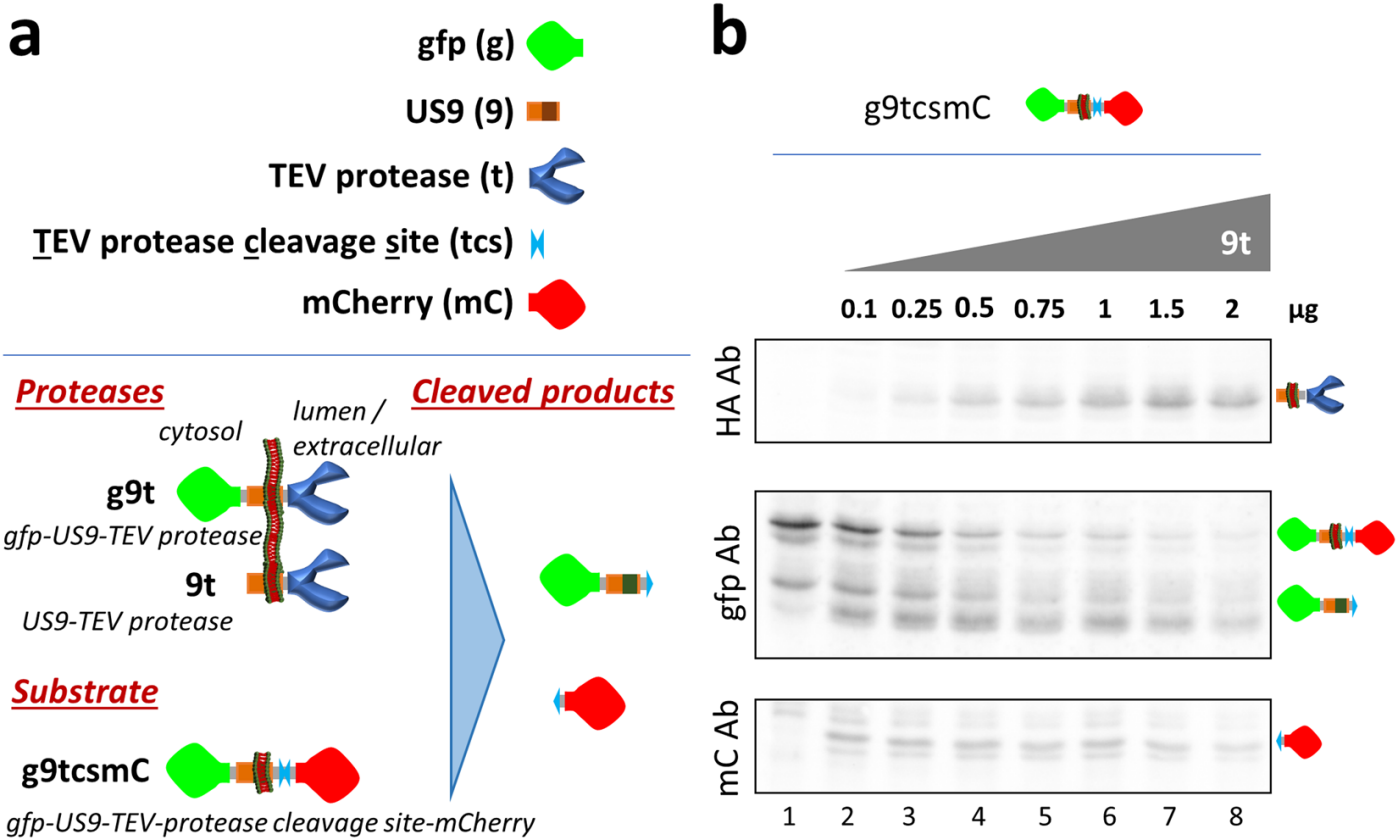
The US9-driven functional assay. (**a**) Schematic representation of elements used to generate the US9 functional assay. The elements were assembled as depicted in the bottom part of the cartoon on the left, with the expected cleaved product on the right. (**b**) Electrophoretic analysis of proteins extracted from 293T cells co-transfected with a constant amount of the substrate g9tcsmC and increasing amounts of the protease 9t, ranging from 0 to 2 μg. The same membrane was incubated with antibodies against HA, gfp, and mCherry to reveal the presence of the effector protein (9t), N-terminal cleaved product (gfp), and C-terminal cleaved fragment (mCherry), as visually represented on the right. The full-length blots are presented in the Supplementary material (S6).

For the purpose of developing a functional assay, we utilized the 27KDa catalytic domain of the Nuclear Inclusion a (NIa) protein from the Tobacco Etch Virus (TEV)^48,49^. This enzymatic activity has been well-characterized in TEV and is not present in mammalian cells^50^, providing a very clean system to detect US9-driven activities. The short cleavage site recognized by the TEV protease is not targeted by other endogenous cellular enzymes, linking the generation of a cleaved product to the presence and close proximity of exogenous protease and substrate. The US9-driven functional assay was assembled as described in Fig. 3a. It is composed of two different parts; a US9-driven TEV protease, and a US9-driven substrate. The US9-driven TEV protease (named 9t) was constructed by attaching the TEV protease to the US9 C-terminus. As a backbone for the substrate we used gfp-US9-mCherry, modified by inserting the TEV protease cleavage sequence (tcs) between US9 and mCherry (g9tcsmC). The outcome of effective functional targeting would produce two cleaved products from the TEV substrate parent protein that will be different in size and recognizable with proper antibodies. The system was expressed and analyzed in HEK-293T cells and representative images of the distribution of US9 driven protease and substrate are shown in Figure S2a. The functional targeting of US9-driven enzyme and substrate was tested by analyzing the presence of cleaved products in protein extracts from cells co-transfected with both constructs. As shown in Fig. 3b, the presence of US9-driven protease (9t) in cells expressing US9-driven substrate (g9tcsmC; lanes 2–8) correlates with the appearance of low molecular weight bands detected with proper antibodies (boxes gfp Ab and mCherry Ab). The same bands were absent from cell lysates in which only the substrate was present (lane 1). The larger cleaved products detected with the gfp antibody is composed of the N-terminal portion of the substrate, containing gfp fused to US9 (US9 yields multiple bands in polyacrylamide gels, probably due to post-translational modifications). The smaller fragment corresponds to the residual C-terminal mCherry. The appearance of the cleaved fragments correlates with the reduction of the full-length uncleaved substrate and with increased expression of TEV protease (Fig. 3b, box HA Ab). The efficiency of the US9-driven functional assay we built was around 70%. To account for possible variability in proteins expression due to different transfection efficiency across samples, we defined the total amount of available substrate as the sum of uncleaved and cleaved fragments, and used these values measured in each individual sample to calculate the percentage of cleavage as follows; cleaved/(cleaved + uncleaved)*100 (Figure S2b). Incidentally, the ability of US9 to drive a functional cargo is not restricted to HEK cells, as indicated by preliminary studies in human osteosarcoma and breast cancer cells (not shown).

**Figure 4:**
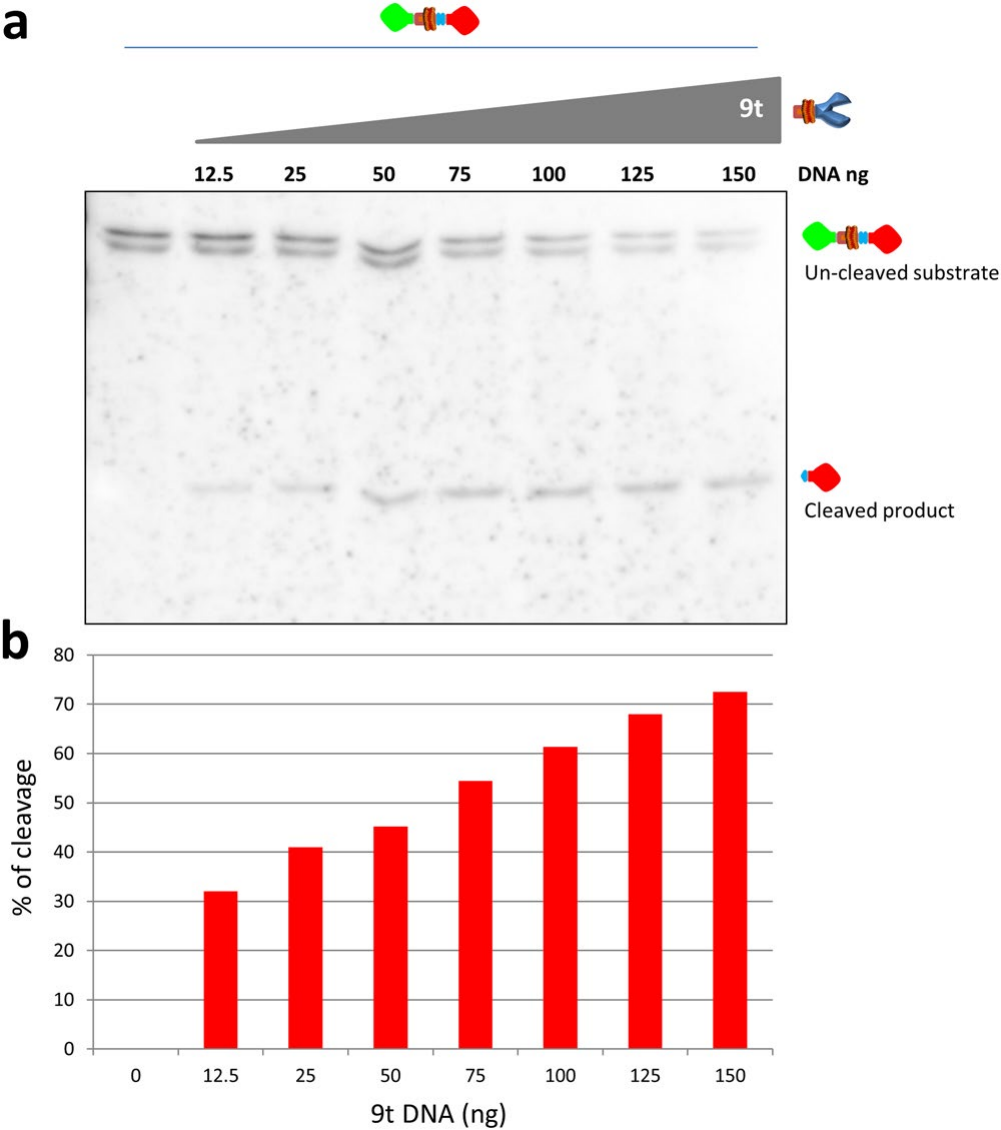
The US9-driven TEV protease expression correlates with substrate processing. (**a**) Electrophoretic analysis of proteins extracted from 293T cells co-transfected with a constant amount of the substrate g9tcsmC and increasing amounts (ranging from 0 to 150 ng) of the protease 9t. The presence of uncleaved and cleaved substrate was revealed with the anti-mCherry antibody. In this range of expression, the increasing presence of the US9-driven protease correlates with a constant decrease of the full length substrate and the corresponding increase of the processed fragment. The densitometric analysis of the same blot was used to generate the histogram shown in (**b**). The height of the columns corresponds to the % of cleavage in each individual sample, calculated as described in Results and in the legend to Figure S2b. The full-length blot is presented in the Supplementary material (S7).

To further assess the capability of the US9-driven functional assay, the experiment was repeated with a lower range of protease expression. Cells were transfected with a constant amount of substrate and with increasing amounts of US9-protease DNA (9t) ranging from 0 to 150 ng. As expected, no cleaved fragments were produced in the absence of the protease (first lane in Fig. 4a). The increasing expression of US9-driven TEV protease caused a corresponding increase in the cleavage of the substrate, clearly detected in lanes 2 (12.5 ng 9t) through 8 (150 ng 9t). The quantitative analysis of the US9-driven activity (Fig. 4b), calculated as explained above, showed a direct correlation between the expression of the protease in this range and the amount of cleaved product. In summary, the US9-driven cleavage assay we generated demonstrates the ability of US9 to target a functional activity to specific cellular microdomains in a stringent and measurable way.

### US9-driven enzymes are active in the vesicle lumen and observe membrane leaflet specificity

**Figure 5:**
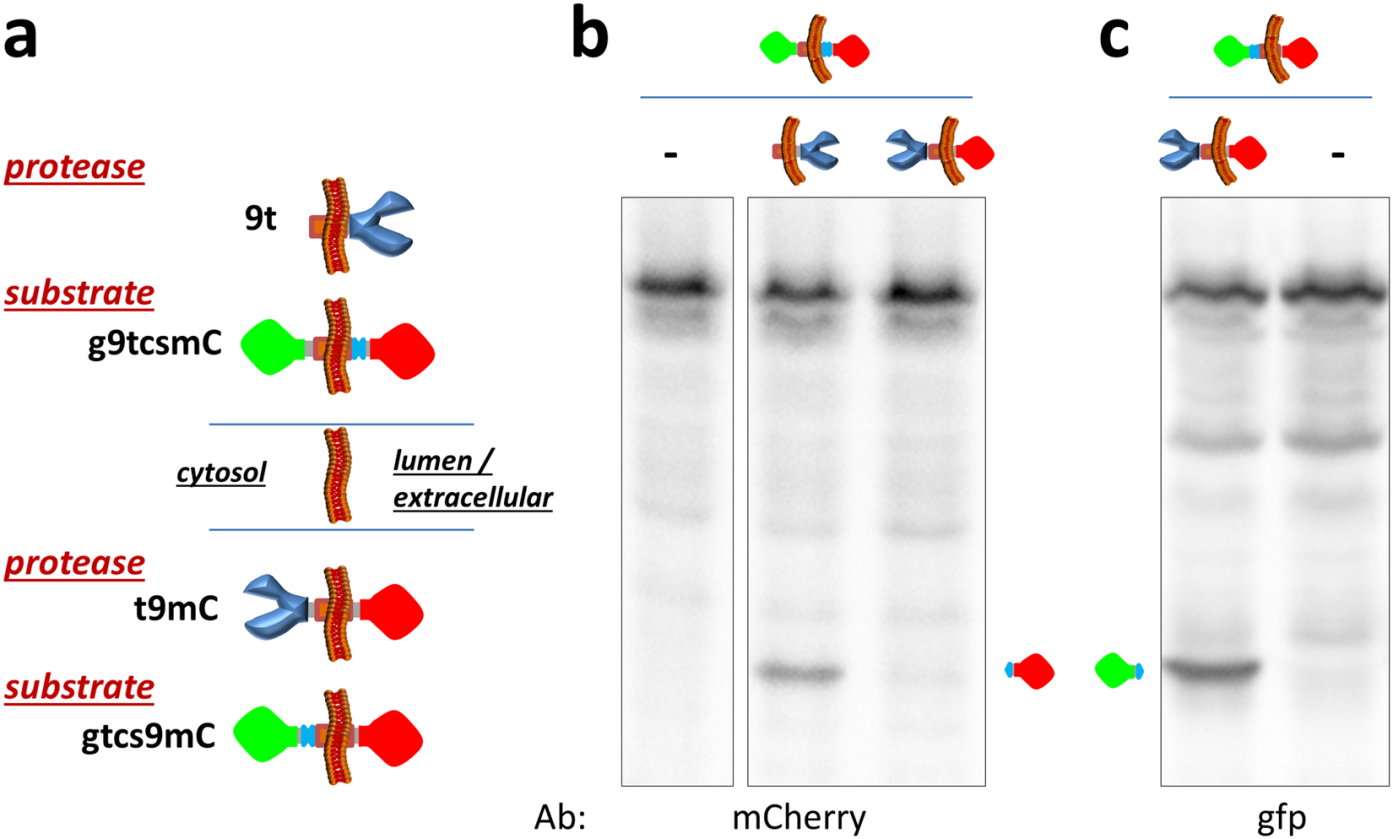
Membrane orientation dependence of the US9-driven functional assay. Novel US9-driven protease (t9mC) and substrate (gtcs9mC) were generated as schematically represented in (**a**) bottom part. In the new constructs, the cargos are attached to the N-terminus of US9 and are exposed on the cytosolic leaflet of membranes, resulting in an inverted orientation with respect to 9t and g9tcsmC. (**b**) Electrophoretic analysis of proteins extracted from 293T cells transfected as indicated. The substrate with TEV protease cleavage site confined in the vesicle lumen is accessible to 9t but not to t9mC, as revealed by the absence of the specific band reacting with the anti-mCherry antibody in the lane corresponding to cells co-transfected with g9tcsmC and t9mC. (**c**) The same cytosol-oriented protease is effective against the substrate with the cleavage site on the cytosolic leaflet of vesicle membranes, and the corresponding gfp product can be seen in cells co-transfected with t9mC and gtcs9mC. The full-length blots are presented in the Supplementary material (S8).

Similar to our evidence indicating that the membrane leaflet orientation of fused fluorescent proteins is determined by their addition to the C- or N-terminal of US9 (Figs 1 and 2), sequence-based structural prediction support the conclusion that molecules fused to the C-terminal domain of US9 are exposed on the extracellular leaflet of the plasma membrane or in the lumen of intracellular vesicles. The effective cleavage of the US9-driven substrate by the protease depends on the close proximity and proper orientation of the two molecules. We wanted to further confirm that the co-presence of both substrate and protease in the same compartmentalized regions (lumen of transport vesicles or extra-cellular space) dictates functional activity. We reasoned that the presence of the enzyme close to its substrate but exposed on the cytosolic side of vesicles would be ineffective toward the US9-driven substrate (g9tcsmC) if the cleavage site is compartmentalized in the vesicle lumen. Therefore, we generated a novel US9-driven protease by replacing gfp with the TEV protease in the gfp-US9-mCherry construct (Fig. 5a) and named it t9mC. In this orientation, the protease will hang on the cytosolic leaflet of vesicles and plasma membranes. The same substrate previously used in the experiment shown in Figs 3 and 4 (i.e. with the cleavage site inside the lumen of the vesicle) was transfected alone or together with US9-TEV protease or TEV protease-US9-mCherry, and the samples were analyzed for the presence of a cleaved product (Fig. 5b). As expected, the enzyme was active toward its substrate when they were brought in close proximity by the targeting properties of US9 and both oriented in the same way, i.e. exposed in the vesicles lumen. The protease oriented in the opposite manner - though still properly targeted by US9 - was completely ineffective and no cleaved fragment could be detected in cells co-transfected with g9tcsmC and t9mC. As a control for the correctness of the constructs used here, we also tested the functionality of the US9-driven, cytosol-oriented TEV protease toward a US9-substrate with the cleavage site exposed on the same side of vesicles membranes (as visually represented in Fig. 5a). The results of this experiment, shown in Fig. 5c, confirmed the ability of the enzyme to cleave a substrate that is both close and exposed on the same leaflet of membranes. Therefore, both close proximity and correct membrane orientation are achieved by the US9-driven functional assay we designed. Based on our experimental approach (analysis of cellular extracts) this conclusion should be for now restricted to intracellular events. We predict that a similar activity occurs at the plasma membrane, but the extracellular products of the US9-targeted function were not analyzed in this study.

### The US9-driven TEV protease activity occurs in lipid rafts

The US9-dependent transport of virions or viral components in the context of viral infection relies on its ability to associate with lipid rafts^43^. For US9 to be used as an effective targeting tool, the chimeric molecules we generated should functionally target lipid raft associated molecules. As a way to test that US9 functionally localizes to relevant lipid raft-associated proteins, we used the US9-driven protease from our functional assay in combination with protease substrates driven by the transmembrane domains of proteins involved in APP processing (i.e. APP and BACE1). APP processing to Aβ through sequential cleavage of APP is modulated by dynamic association with lipid rafts^10^, and is a pathway highly relevant to pathology in neurons^51–53^.

**Figure 6:**
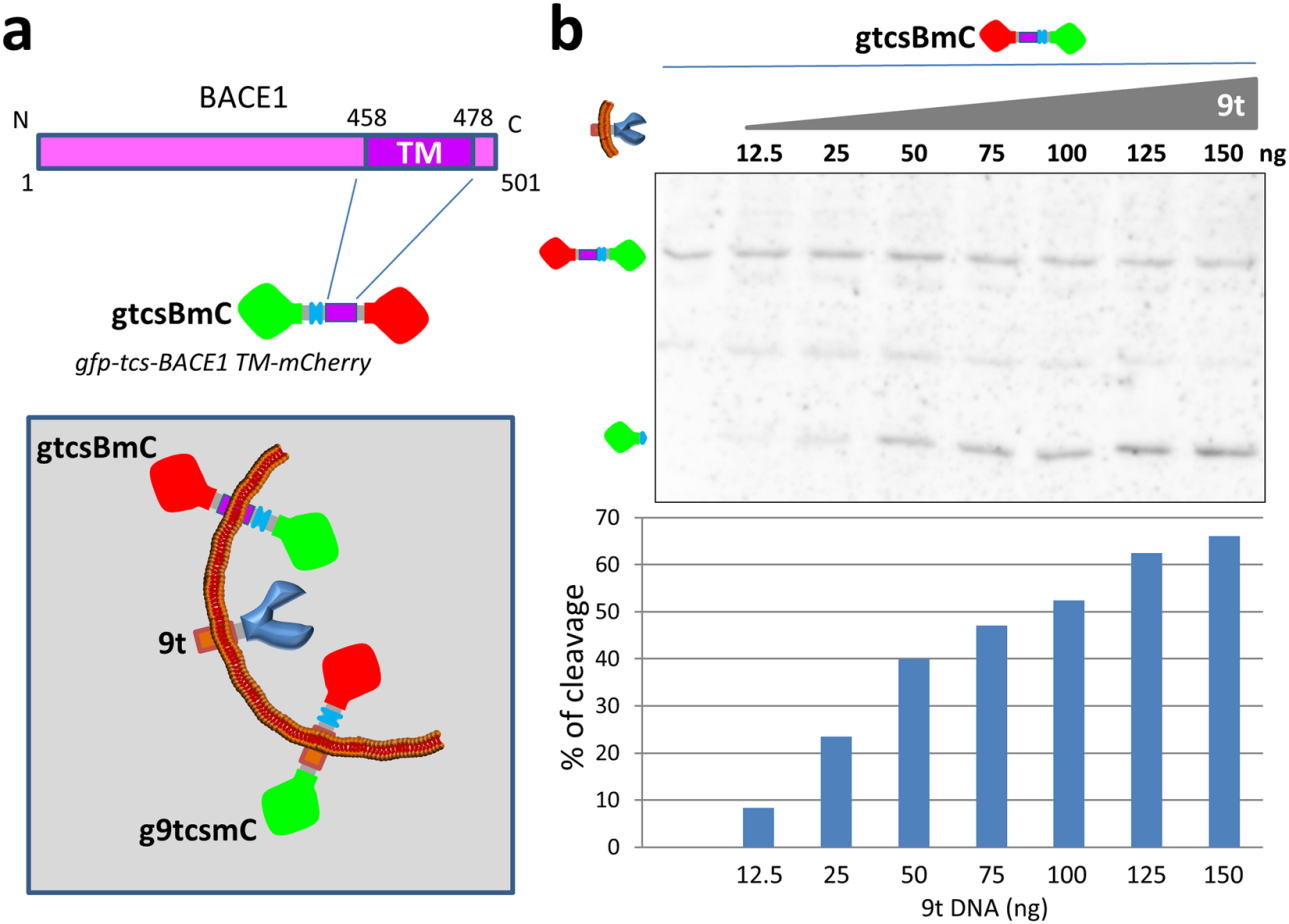
BACE1-based substrate for US9-driven functional assay. The sequence encoding amino acids 454–481 of BACE1, comprising the trans-membrane domain, was used to generate the US9 functional assay substrate gtcsBmC. In this construct, the TEV cleavage site is inserted between gfp and BACE1-TM, as represented in the cartoon in (**a**). BACE1 is type 1 membrane protein, with the C-terminus exposed on the cytosolic side of membranes. The membrane orientation of the BACE1-based substrate, with respect to the other elements of the US9 functional assay, is schematically visualized in the boxed cartoon. In 293T cells expressing constant amounts of gtcsBmC, increasing presence of 9t results in the appearance of a cleaved product, revealed by the band corresponding to gfp in the electrophoretic analysis in (**b**). Cleavage efficiency determined as already described correlates with the amount of 9t DNA transfected in each individual sample and is graphically rendered in the chart. The full-length blot is presented in Supplementary material (S9).

Briefly, APP can be processed by a β-secretase, BACE1, to produce a fragment that can then be cleaved by a γ-secretase to release Aβ. APP cleavage by BACE1 is thought to occur mainly in lipid rafts and we selected BACE1 as an indicator of the US9-driven functional ability to affect lipid rafts molecular events^10,54^. Hence, we modified the US9-based substrate g9tcsmC by replacing the US9 domain of the chimeric protein with the trans-membrane domain of BACE1 (BACE1-TM)^55^, to determine if it was accessible to the activity of the US9-driven protease. Transmembrane domains play a critical role in proper membrane partitioning and proteins sorting/localization^35–39^. Unlike US9, BACE1 is a type I membrane protein, with the C-terminal portion exposed on the cytosolic side of membranes. Therefore, the orientation of the cleavable reporter was inverted, with the TEV protease cleavage site inserted between gfp and BACE1-TM, to generate gtcsBmC, as described in Fig. 6a. Cells were co-transfected with the BACE1-based substrate and increasing amounts of the US9-driven protease, and the activity of the enzyme was assessed. As shown in Fig. 6b, no cleavage occurred in the absence of the protease. When the enzyme was co-expressed with the substrate, a cleaved fragment was readily produced, in a US9-protease expression dependent manner, as quantified in the histogram of Fig. 6b. The results of this experiment led us to conclude that a BACE1-based substrate is accessible to the activity of the US9-driven protease.

**Figure 7:**
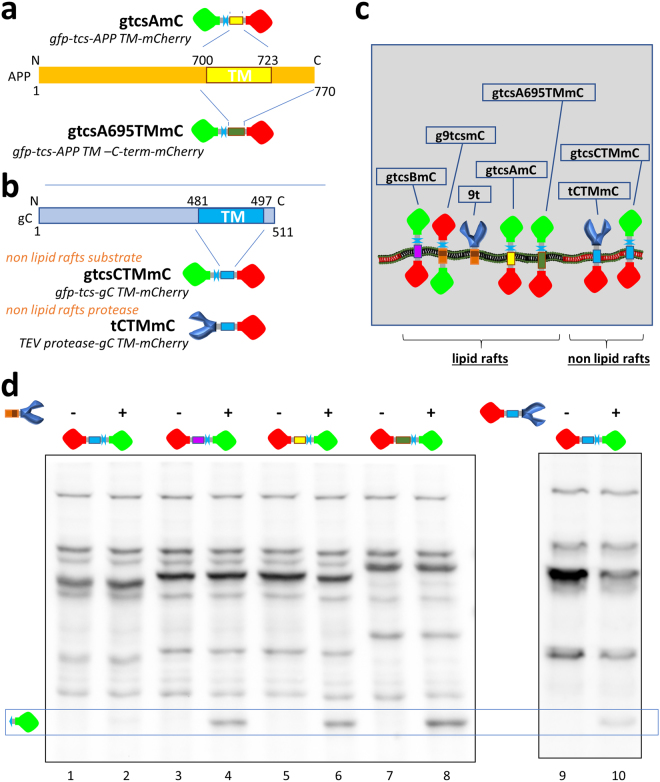
US9-driven TEV protease activity on lipid raft and non lipid raft substrates. (**a**) Two APP-based substrates were generated as depicted, by inserting the APP TM domain (aa 700–723) into the substrate backbone. In gtcsAmC, the TEV protease cleavage sequence (tcs) is upstream of aa 694–729 of APP. In gtcsA695TMmC the APP sequence starts from amino acid 694 and goes to the C-terminus of APP, and comprises trans-membrane and cytosolic domains. The APP α-cleavage site (aa 688) is not present in the constructs. Canonical APP770 aa numbering is used here. (**b**) Non-lipid raft substrate and protease were based on the HSV glycoprotein C TM domain (aa 481–497), as schematically represented. (**c**) The orientation and lateral membrane organization of generated substrates and proteases is represented in the cartoon. US9 is a type 2, while BACE1, APP, and gC are type 1 membrane proteins. All constructs are designed and assembled in order to expose cleavage site and protease in the lumen of transport vesicles. Moreover, lateral proximity dependent on accumulation in lipid rafts is visually represented in the cartoon. (**d**) The ability of 9t to cleave substrates driven by the different TM domains indicated in (**c**) was determined in 293T cells. Targeting domains derived from BACE1 (lanes 3–4) and APP (lanes 5–6 for TM and 7–8 for TM + cytosolic domain) were all able to drive the TEV protease cleavage site in close proximity of 9t, resulting in effective processing as demonstrated by the appearance of the band corresponding to gfp (boxed region of the western blot). The substrate driven by gC TM domain was unaffected by the co-expression of 9t, as no difference is detectable between lanes 1 (gtcsCTMmC) and 2 (gtcsCTMmC + 9t). The same substrate was readily cleaved in cells co-transfected with the gC TM-targeted protease (lanes 9–10). The full-length blots are presented in the Supplementary material (S10).

The other key component of the APP processing is obviously APP itself. APP cleavage is a complex regulated process in which lipid rafts association seems to play a critical role. Attracted by the possibility that we may eventually modify the molecular machinery responsible for Aβ production, we further investigated the ability of US9 to functionally act on APP-based substrates. APP is a type I membrane protein, and therefore we used gtcsBmC as a backbone to replace the BACE1-TM with the APP trans-membrane domain, without or with the APP C-terminal domain. The two new substrates were named gtcsAmC and gtcsA695TMmC, respectively, and their organization with respect to the APP sequence is exemplified in Fig. 7a.

To add further evidence that US9 fusion proteins localize specifically with other lipid raft proteins, we performed additional functional experiments using glycoprotein C (gC) from HSV 1. The gC is a type I membrane protein, similarly to BACE1 and APP, that does not localize to lipid rafts, and its trans-membrane domain (gC-TM) has been used in chimeric reporters to show non lipid raft localization^56^. As shown in Fig. 7b, we inserted the gC-TM domain to replace the BACE1-TM in the BACE1-based substrate, and the resulting gC-based substrate gtcsCTMmC was used in US9-driven functional assay (please see Figure S3 for the gtcsCTMmC’s sucrose gradient validation). Finally, as a control for a non-lipid raft activity, we attached the TEV protease to the same gC-TM domain, and generated tCTMmC. The predicted localization of the polypeptides with respect to lipid rafts association is summarized in Fig. 7c.

These constructs were then tested for their ability to act as substrates for the activity of the US9-driven protease (Fig. 7d). The gC-driven substrate was not cleaved when co-expressed together with 9t, implying a different membrane localization for the two proteins. On the other hand, substrates driven by BACE1-TM, APP-TM, or APP695TM could all interact with the protease, and the effect of this interaction resulted in the release of the cleaved fragment, detected with the gfp antibody in Fig. 7d (boxed bands). The non-lipid rafts gC-TM-driven substrate was cleaved in the presence of the gC-TM-driven protease, demonstrating that the absence of processing in cells co-expressing gtcsCTMmC and 9t was dependent on the different membrane localization of the two molecules.

All together data from this set of experiments support the association of the US9-driven functional cargo with lipid rafts and its ability to modify the APP molecular machinery.

## Discussion

Membrane microdomains (e.g. lipid rafts) and compartmentalization play a major role in numerous physiological and pathological processes, including HIV infection. Lipid rafts were recently suggested to also contribute to HIV neuropathogenesis^23–25^. However, due to the limited availability of specific study tools, the exact contribution of lipid rafts to HAND is still unclear. Here we present evidence that the lipid-raft dwelling protein US9 can be used to investigate the contribution of lipid rafts-dependent changes induced by viral neurotoxins.

US9 is an exogenous protein, with no known deleterious effect on mammalian cells. We show that US9-driven recombinant cargos can be attached to either terminus of US9 without altering its cellular distribution. This first finding extends previous observations regarding stand-alone properties of US9 and provides a basis for the further use of US9 in functional assays whereby the US9’s targeting capabilities could be used to specifically target enzymatic machinery to lipid raft microdomains. Of note, US9 distribution/trafficking behavior is maintained in different cell types, both primary cells (neurons) and various cell lines^32^. The US9-driven functional assay we introduce here is based on the constitutively active exogenous proteolytic enzyme TEV protease. Interactions between the US9-guided exogenous protease and recombinant proteins containing its cleavage sequence occurred predictably and relied on both protease and substrate sharing similar cellular compartmentalization, lateral membrane segregation, and membrane leaflet orientation. The observed cleavage of the substrates by the US9-driven protease can only result from the close proximity of the two molecules. Therefore, cleavage represents the evidence of effective enzymatic processing, and provides corroborative functional evidence of lipid-raft determination by biochemical means.

The US9-based functional assay we designed and tested was instrumental in demonstrating the ability of US9 to target a functional cargo to the luminal leaflet of cellular vesicles, and to actively target molecules involved in APP processing. For this reason, we selected an exogenous proteolytic activity not present in mammalian cells, in order to provide unambiguous results exclusively dependent on the effect of the protease on its specific substrate. Nevertheless, based on our data, the US9 targeting capabilities can be used to directly manipulate the APP processing machinery, to help understand the contribution of individual steps in neuronal alterations depending on the presence of viral neurotoxins. Additionally, fluorescently tagged US9 proteins such as g9, 9mC, and g9mC can provide a snapshot of targeted interaction/cleavage. This may have important consequences for studies aimed at exploring the role of trafficking alterations in the regulation of APP processing by HIV neurotoxins^22,23,25^. Overall, our data and the described US9 physiological function/tropism in the context of viral infection support the hypothesis that US9-based molecular tools would also work in more natural conditions (i.e. endogenous APP processing in neurons) – ongoing studies in neurons suggest this to be the case.

Experiments in this study were designed to focus our analysis on intra-cellular events, as intra-neuronal accumulation of Aβ has been widely reported in the HIV-infected brain^25,57^. However, HIV is known to affect amyloidogenesis in different ways^58^, and deposition of Aβ plaques is also often found in aging HIV-infected individuals^59,60^. As both US9 and APP machinery can transiently accumulate on the plasma membrane, US9-dependent functional activity could also be used to study events that occur on the plasma membrane. Further studies are necessary to address this issue and are of great import to HAND.

In conclusion, we propose US9 as a novel tool to assess the contribution of lipid rafts to the neuronal dysfunction caused by HIV neurotoxins or by other insults that are associated with altered protein transport and/or accumulation.

## Materials and Methods

### Cells cultures and transfections

Rat cortical neurons (RNs) were obtained from the brains of 17–18 day old rat embryos and cultured in Neurobasal medium containing B27 supplement (Gibco) as detailed by Sengupta *et al*.^61^ and originally described by Brewer *et al*.^62^.

For transfections, RNs were seeded on poly-L-Lysine coated glass coverslips (35,000 cells/coverslip) in Neurobasal/B27 medium containing 2% Horse serum for 2 hours. Coverslips were then washed and media replaced with Neurobasal/B27 media containing GlutaMAX™, Glutamic Acid and Gentamycin. After 4 days, fresh medium not containing L-Glutamic acid was used to replace old medium. At 5 days *in vitro* (DIV5), half volume was removed and stored at 37 °C, and replaced with fresh medium. After 6 hours, the medium was reduced to 0.6 ml and the removed volume pooled with the previously stored medium to make conditioned medium. Lipofectamine 2000 (Invitrogen) – DNA complexes (2 μl of Lipofectamine 2000 per transfection) were prepared in OptiMEM (Gibco) according to manufacturer directions, incubated for 5′ at room temperature in the dark, and transferred dropwise on coverslips. After 1 hour incubation at 37 °C, transfection medium was removed and replaced with conditioned medium.

Human Embryonic Kidney Cells (HEK) 293T were grown in Dulbecco Modified Eagle Medium (DMEM) supplemented with 10% Fetal Calf Serum (FCS), and Gentamycin. For transfections, cells were seeded on 12 well plates, 160,000 cells/well, the day before treatment. Lipofectamine 2000 – DNA complexes were prepared and added to cells as described above for RNs, and incubated for 2.5 hours before replacement with fresh medium. For co-transfections with variable amounts of plasmids, the total amount of DNA in each sample was kept constant by adding the empty pcDNA3.1 vector.

### Ethics Statement

Animals were used as a source of brain tissue to prepare neuronal cultures, following the recommendations in the Guide for the Care and Use of laboratory Animals of the National Institute of Health. This protocol for harvesting brain tissue was approved by the Institutional Animal Care and Use Committee of Drexel University (PHS Animals Welfare Assurance #A-3222-01), approved on 09-18-2015 (permit #20439).

### Fluorescence microscopy analysis and immunodetection

Cells were fixed in 4% paraformaldehyde 24 hours post transfection. For permeabilization, fixed cells were incubated with Phosphate Buffered Saline (PBS) containing 0.2% Triton X100, 1% Bovine Serum Albumine (BSA), 0.5% FCS for 30 minutes at Room Temperature. Non permeabilized cells were incubated with the same buffer without Triton X100. Permeabilized and non permeabilized cells were first incubated with the indicated antibodies for 40′ at RT, and subsequently with the appropriate secondary antibodies for 20′ at RT. Nuclear counter-staining was done via incubation of fixed cells with Hoechst 33342 (Molecular Probes - Invitrogen). Primary antibodies used for immunodetection were the anti-mCherry Rabbit polyclonal Antibody from Abcam (ab 183528), 1:1000 dilution, and the anti-Hemagglutinin (HA) mouse monoclonal antibody from Santa Cruz Biotechnology (sc-7392), 1:500 dilution. Secondary antibodies (Life Technologies) were Alexa Fluor® 488 goat anti-rabbit for detection of mCherry, and Alexa Fluor® 568 goat anti-mouse for detection of HA.

Confocal images were taken under a Zeiss Axio Imager.Z1m microscope equipped with Plan-apochromat 63x/1.4 oil DIC objective. Acquisition software was the provided AxioPlan LSM 510. All images were analyzed and assembled using Fiji. Images are representative of the original data and comply with the journal image processing policy.

### Protein electrophoretic analysis and western blotting

Cells were harvested and lysed in RIPA buffer (150 mM sodium chloride, 1% Triton X-100, 0.5% sodium deoxycholate, 0.1% SDS (sodium dodecyl sulfate), 50 mM Tris, pH 8.0) containing proteases/phosphatases inhibitors for 15′ on ice with occasional vortexing, and centrifuged for 10′ at 4 °C at 10,000 rpm. Protein extracts in the supernatants were separated on denaturing 10% polyacrylamide gels (SDS Page) and transferred to a PVDF membrane for immunoblotting. Primary antibodies used were: anti-mCherry Rabbit polyclonal from Abcam (ab 183528), 1:2000, anti-HA mouse monoclonal from Santa Cruz Biotechnology (sc-7392), 1:1000, anti-gfp Rabbit polyclonal from Santa Cruz Biotechnology (sc-8334), 1:500, anti-human Transferrin Receptor (TfR) mouse monoclonal from Invitrogen (13-6800), 1:1000. Secondary antibodies and detection reagents were from Pierce (SuperSignal West Femto kit).

### Lipid raft flotation assay

Identification of lipid rafts through flotation on OptiprepTMsucrose gradient is a standard procedure, widely described in the literature^44–46,63^. Here we followed the protocol used to study the association of PRV US9 with lipid rafts from infected cells^43^.

Briefly, cells were harvested, washed once on ice in PBS, lysed in 1 ml ice cold lysis buffer (1% Triton X100 in TNE: 25 mM Tris pH 6.8, 150 mM NaCl, 5 mM EDTA) with proteases/phosphatases inhibitors, homogenized by passing 15 times through an 18-gauge needle, rocked for 30′ at 4 °C, homogenized again (5 times through an 18-gauge needle), and finally mixed with 2 ml of ice-cold 60% OptiprepTM density gradient medium (Sigma-Aldrich). The gradient was prepared by placing the cells homogenate at the bottom of a Beckman SW41 ultracentrifuge tube and subsequently overlaying it with 5 ml of ice-cold 30% Optiprep in TNE and 4 ml of ice-cold 5% Optiprep in TNE. Samples were centrifuged at 34,200 rpm (200,000 × g) at 4 °C for 20 hours. 1 ml fractions were collected from top and analyzed by SDS Page.

### Plasmid construction

A complete list of plasmids generated in this study is provided in Figure S4. In all constructs, protein expression is driven by the CMV promoter present in the original pEGFP-C1 vector (Clontech) that was used to create gfp-US9 (g9)^32^. Constructs were sequenced and functionally validated.

Restriction enzymes and Q5 High Fidelity DNA polymerase used for cloning were from New England Biolabs. Ligations were performed using DNA Ligation kit ver. 2 from TaKaRa. Oligonucleotides were from IDT.

In order to allow expression of C-terminal fusion proteins, the stop codon at the end of the US9 sequence was removed by PCR in the intermediate plasmid g9nostop. mCherry sequence was amplified from pmCherry-N1 (Clontech) and inserted in g9nostop to generate g9mC. 9mC was constructed by replacing the gfp sequence in the intermediate plasmid 9g with the mCherry sequence from pmCherry-N1.

TEV protease sequence was amplified from plasmid mCherry_LD 0_TEV (a kind gift of Dr. Joshua Leonard - Addgene #58868)^50^ and inserted in g9nostop to generate g9t. 9t was subsequently obtained by collapsing the region comprising gfp in g9t. t9mC was obtained by replacing the gfp sequence in g9mC with the TEV protease amplified sequence. To construct the US9-based TEV protease substrates, oligonucleotides coding the amino acid sequence GSENLYFQ/G were annealed *in vitro* and inserted in g9mC (TEV protease cleavage site is underlined; cleavage occurs between Q/G). Convenient restriction sites were added at the oligonucleotides 5′- and 3′-ends to allow cloning between US9 and mCherry in g9tcsmC, or gfp and US9 in gtcs9mC. DNA sequences coding for BACE1 (aa 454–481) and APP (aa 694–729) regions comprising transmembrane domains were amplified from 293T cells genomic DNA and used to replace the US9 sequence in gtcs9mC, to obtain gtscBmC and gtcsAmC, respectively. To generate gtcsA695TMmC, the sequence encompassing trans membrane and cytosolic domains of APP was amplified from plasmid pCAX APP 695 (a kind gift of Dr. Dennis Selkoe & Tracy Young-Pearse - Addgene #30137)^64^ and inserted into gtcs9mC to replace the US9 sequence. APP amino acids numbers refer to APP770.

To generate gtcsCTMmC - the substrate based on HSV glycoprotein C (gC) trans membrane domain (CTM) - the sequence coding for aa 478–504 (sequence reference GenBank: GU734771.1) of gC was amplified from viral DNA and cloned into gtcsBmC to replace the BACE1 TM portion of the substrate. The CTM-driven TEV protease was obtained by replacing the gfp-tcs sequence in gtcsCTMmC with the TEV protease sequence, and the resulting plasmid was named tCTMmC.

A detailed description of regions present between main elements in all these constructs is given in Figure S4.

### Ethical approval and informed consent

Please see methods section for details about institutional approval (animal protocols only - no humans/human samples were used in this study).

## Electronic supplementary material

Supplementary figures
