# Supplementary material for: The lipid raft-dwelling protein US9 can be manipulated to target APP compartmentalization, APP processing, and neurodegenerative disease pathogenesis
Source: Sci Rep. 2017 Nov 8;7:15103. doi: 10.1038/s41598-017-15128-8 (PMC5678071; doi:10.1038/s41598-017-15128-8)
Supplement: Supplementary file 1 — Supplementary figures [file 41598_2017_15128_MOESM1_ESM.pdf]

The lipid raft-dwelling protein US9 can be manipulated to target APP compartmentalization, APP processing, and neurodegenerative disease pathogenesis

Supplementary Materials

S1-S4: Supplementary Figures and Legends

S5-S10: Full-length blots of experiments shown in main figures

Renato Brandimarti[1,2,*], Gordon S. Hill[1], Jonathan D. Geiger[3], and Olimpia Meucci[1,4,*]

Department of Pharmacology and Physiology[1], and Department of Microbiology and Immunology[4], Drexel University College of Medicine, Philadelphia (PA).

Department of Basic Biomedical Sciences[3], University of North Dakota, School of Medicine and Health Sciences, Grand Forks (ND).

Department of Pharmacy and Biotechnologies[2], Alma Mater Studiorum, University of Bologna, (Italy)

[*] Corresponding authors: Olimpia Meucci and Renato Brandimarti

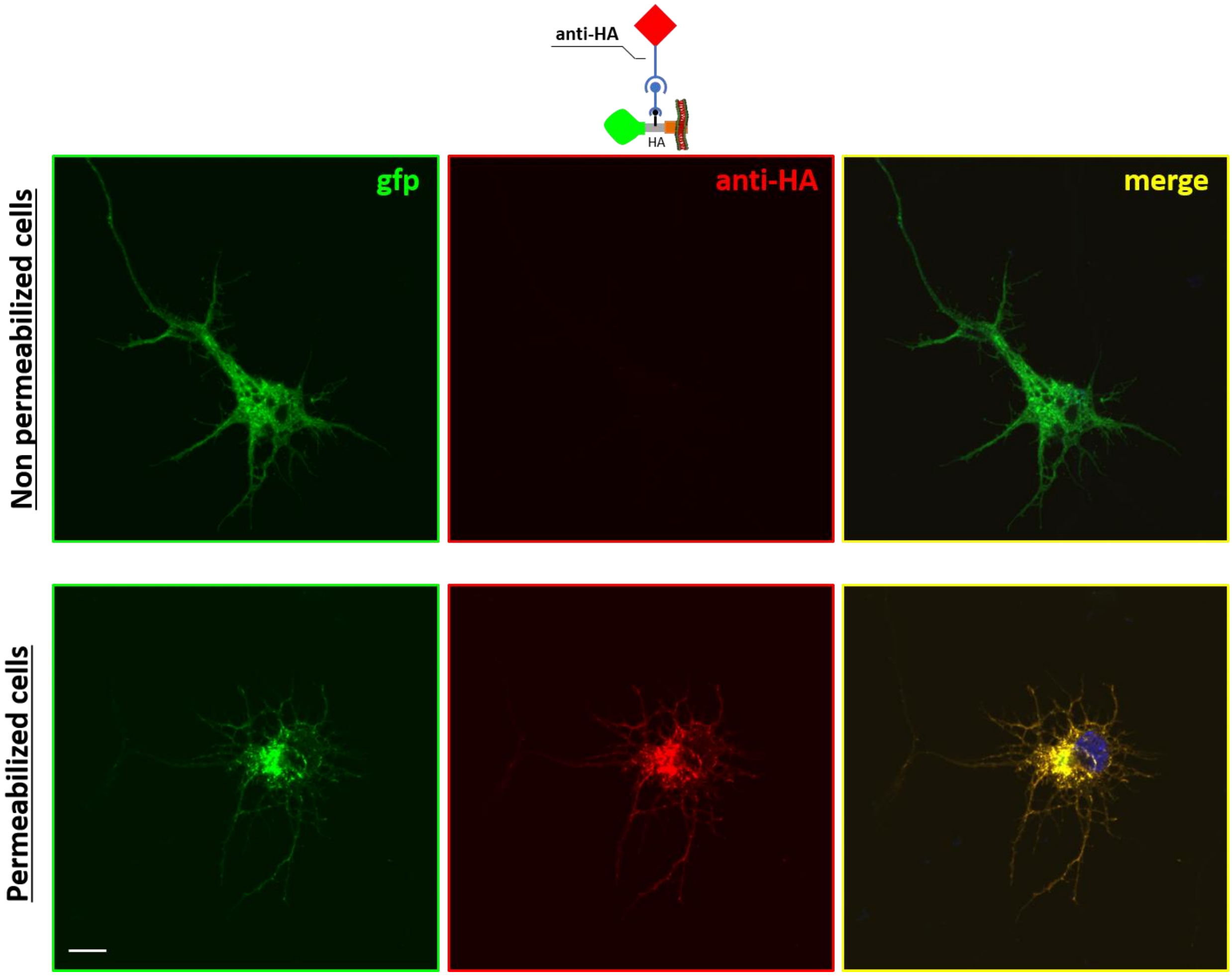

Figure S1. Membrane orientation of US9 N-terminal cargos. Confocal microscopy was used to image rat cortical neurons expressing g9 (green: gfp; red: anti-HA; top row: non-permeabilized neurons; bottom row permeabilized neurons). As shown in the central micrographs, immunodetection of the HA epitope was precluded in non-permeabilized neurons, while the same epitope was readily available in permeabilized cells. Scale bar is 10µm.

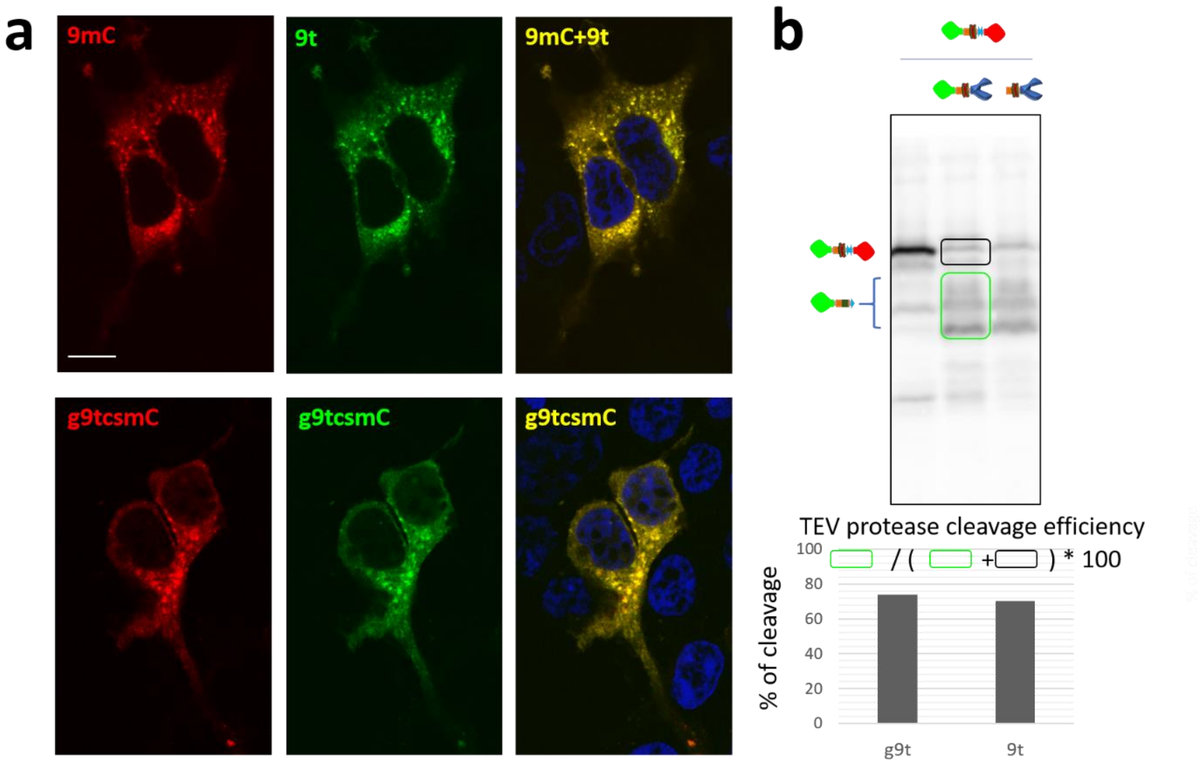

Figure S2. The US9-driven functional assay. a) Representative images of 293T cells co-expressing US9 with C-terminal cargos mCherry (9mC) and TEV protease (9t) are shown in the top panels. The distribution of 9mC is revealed by the mCherry red signal in the left panel, while 9t localization (in green in the center) is detected with an antibody against the HA epitope present in 9t. Merged signal in the right image confirms the co-localization of the two molecules. The distribution of the substrate g9tcsmC is shown in the lower panels (red: mCherry; green: gfp; yellow: merged image). Scale bar is 10µm. **b)** Quantitative analysis of cleavage efficiency. Densitometric quantification of the abundance of bands corresponding to uncleaved (black box) and cleaved (green box) substrate in 293T cells, cotransfected as visually indicated on the top of the western blot, was used to assess the cleavage efficiency of the US9-driven TEV protease. Columns on the bottom chart represent the percentage of processing in each individual sample, calculated as depicted above and explained in Results. The gfp antibody was used for detection. Full-length blot is shown in figure.

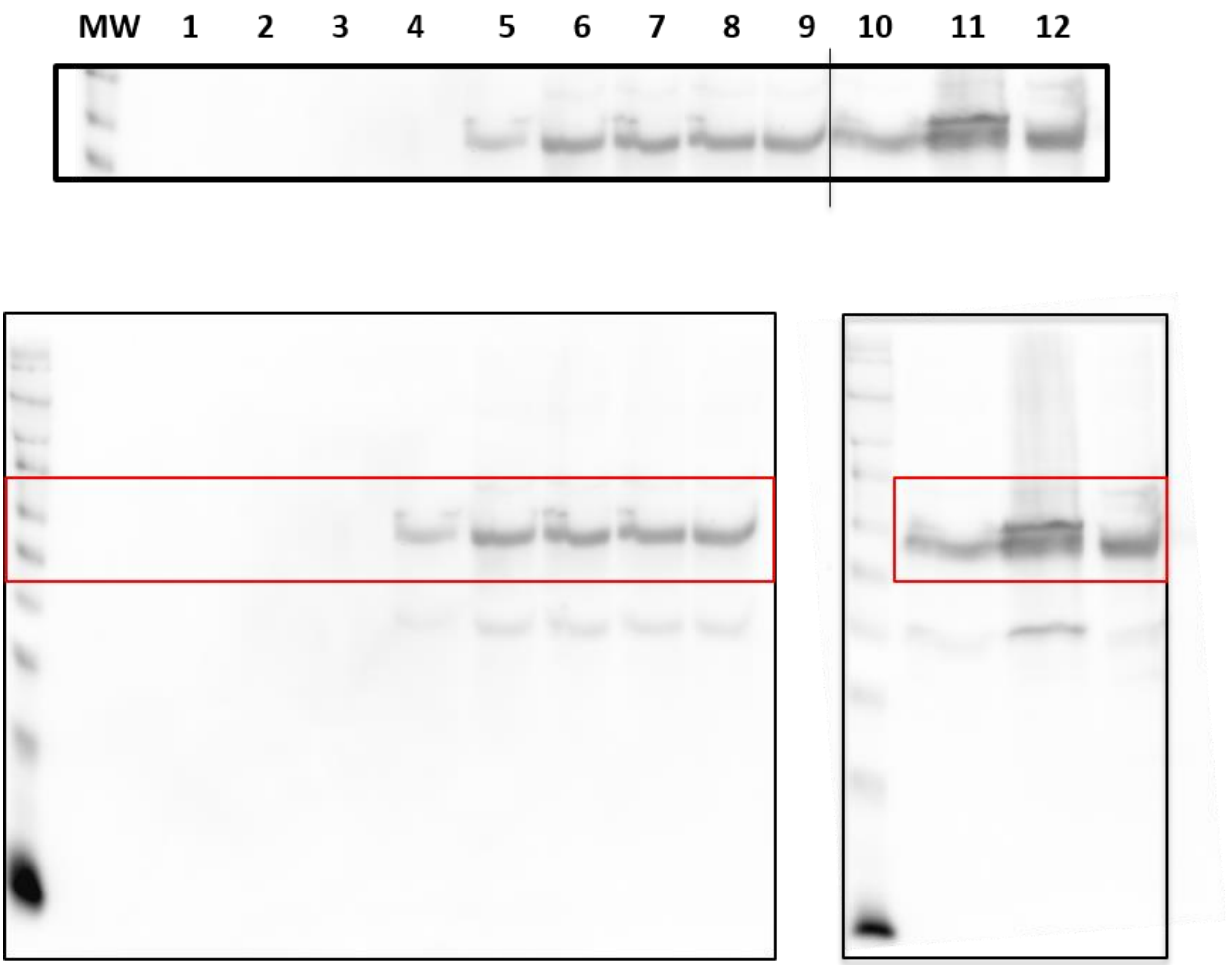

Figure S3. Electrophoretic analysis of proteins from 293T cells expressing gtcsCTMmC, fractionated on a Optiprep gradient as described in the Methods. The presence of gtcsCTMmC was detected on each fraction with an antibody against gfp. Sequential fractions collected from top are loaded in lanes 1-12. Samples were run on two gels, as indicated by the line between lanes 9 and 10 in the first blot (top). The full-length blots are also presented separately below the cropped blot (red boxes mark lanes corresponding to the top blot).

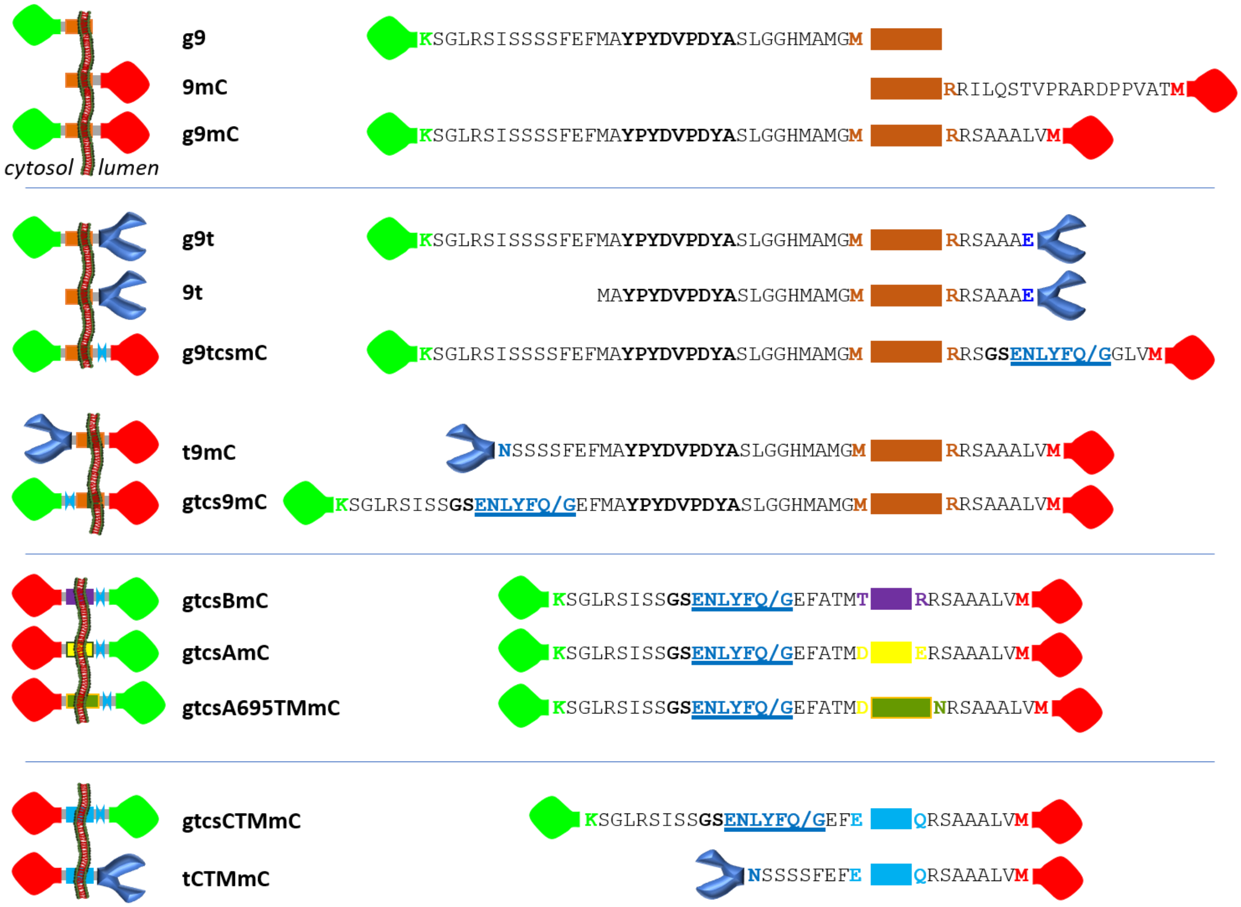

Figure S4. Description of recombinant proteins. With the exception of g9, all other constructs were generated in this study. On the left, the membrane orientation and composition of each recombinant protein is graphically rendered. On the right, the regions joining the different elements in the chimeras (gfp, mCherry, TEV protease, targeting domain) are expanded to show aminoacid composition. The TEV protease cleavage site (tcs) is in bold, underlined light blue. Cleavage occurs between Q and G. The HA epitope is in bold.

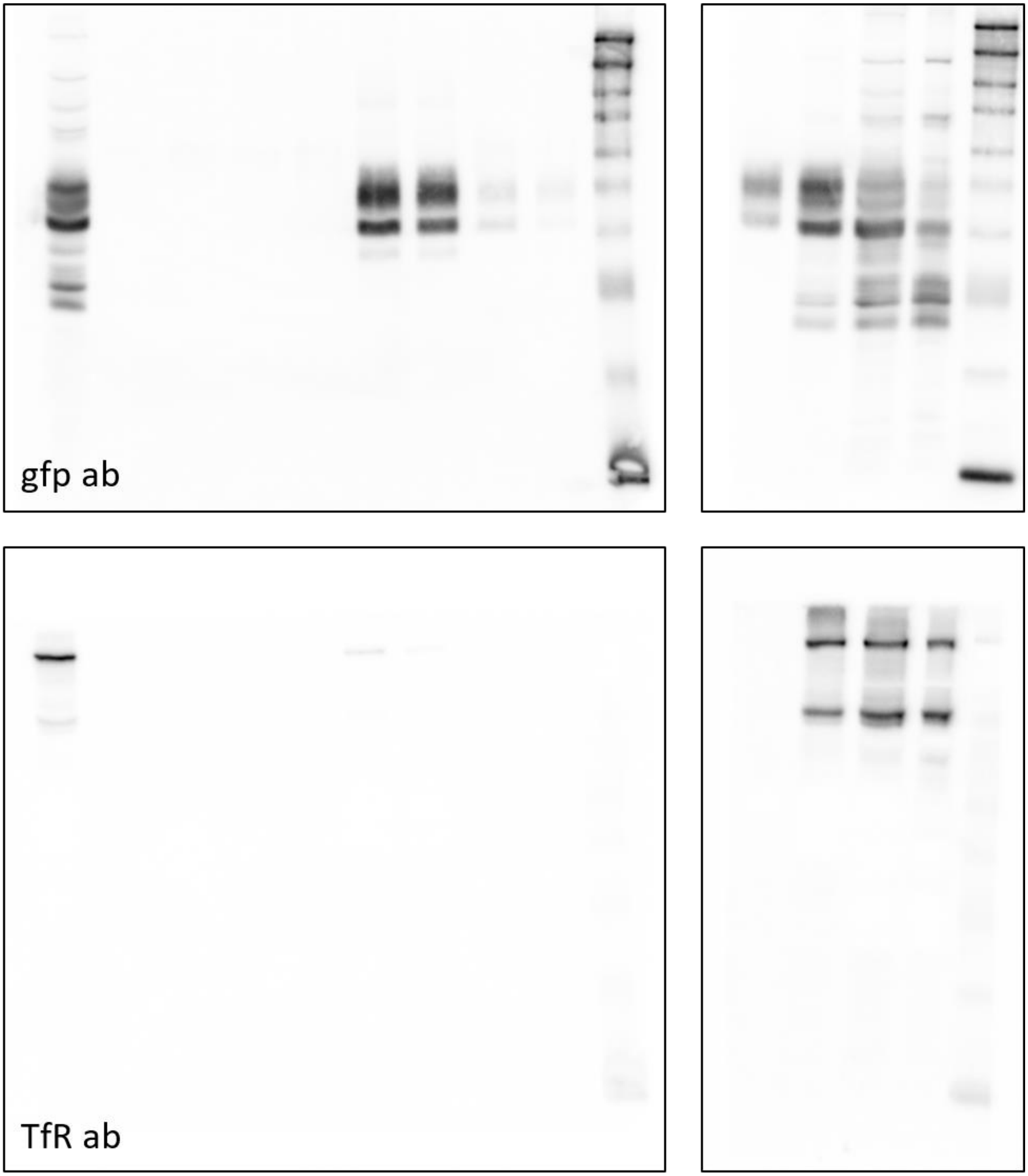

Suppl. material S5: Original blots used to generate Figure 1a

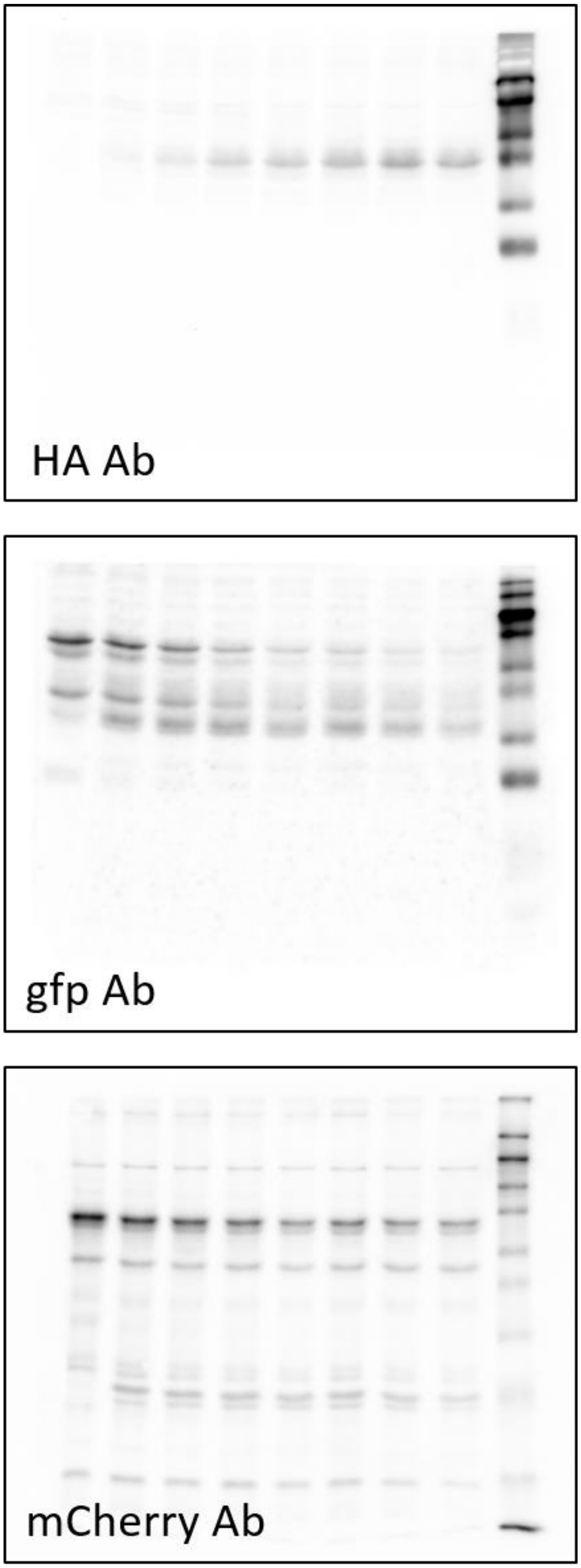

Suppl. material S6: Original blots used to generate Figure 3b

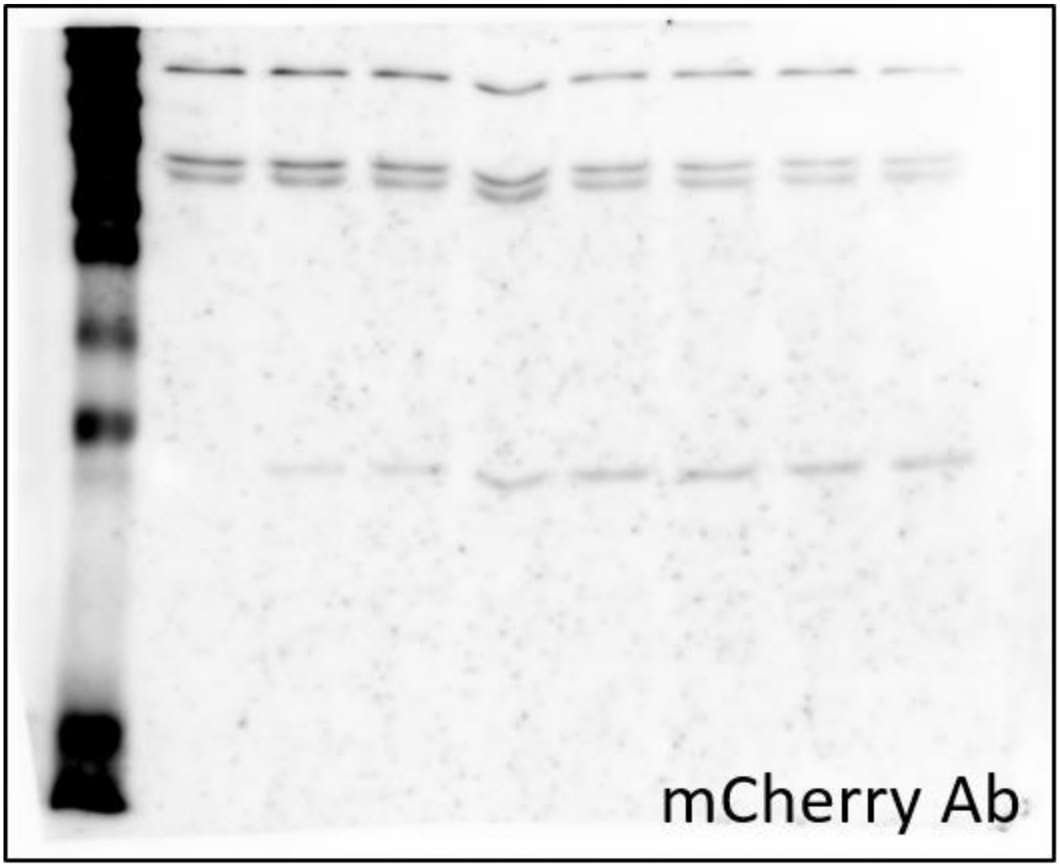

Suppl. material S7: Original blot used to generate Figure 4a

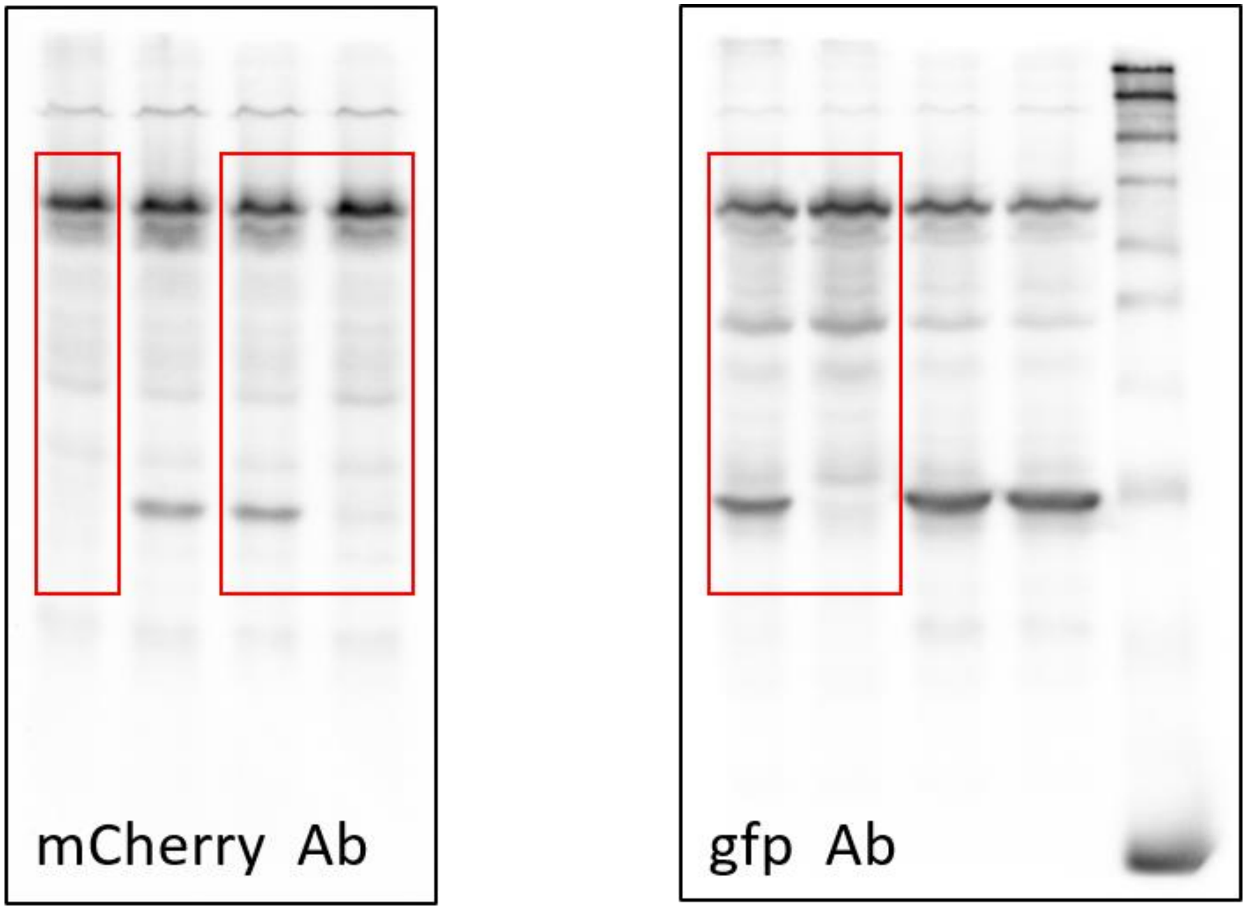

Suppl. material S8: Original blots used to generate Figure 5b and c; red boxes mark the lanes reported in figure 5. The additional lanes contain irrelevant samples.

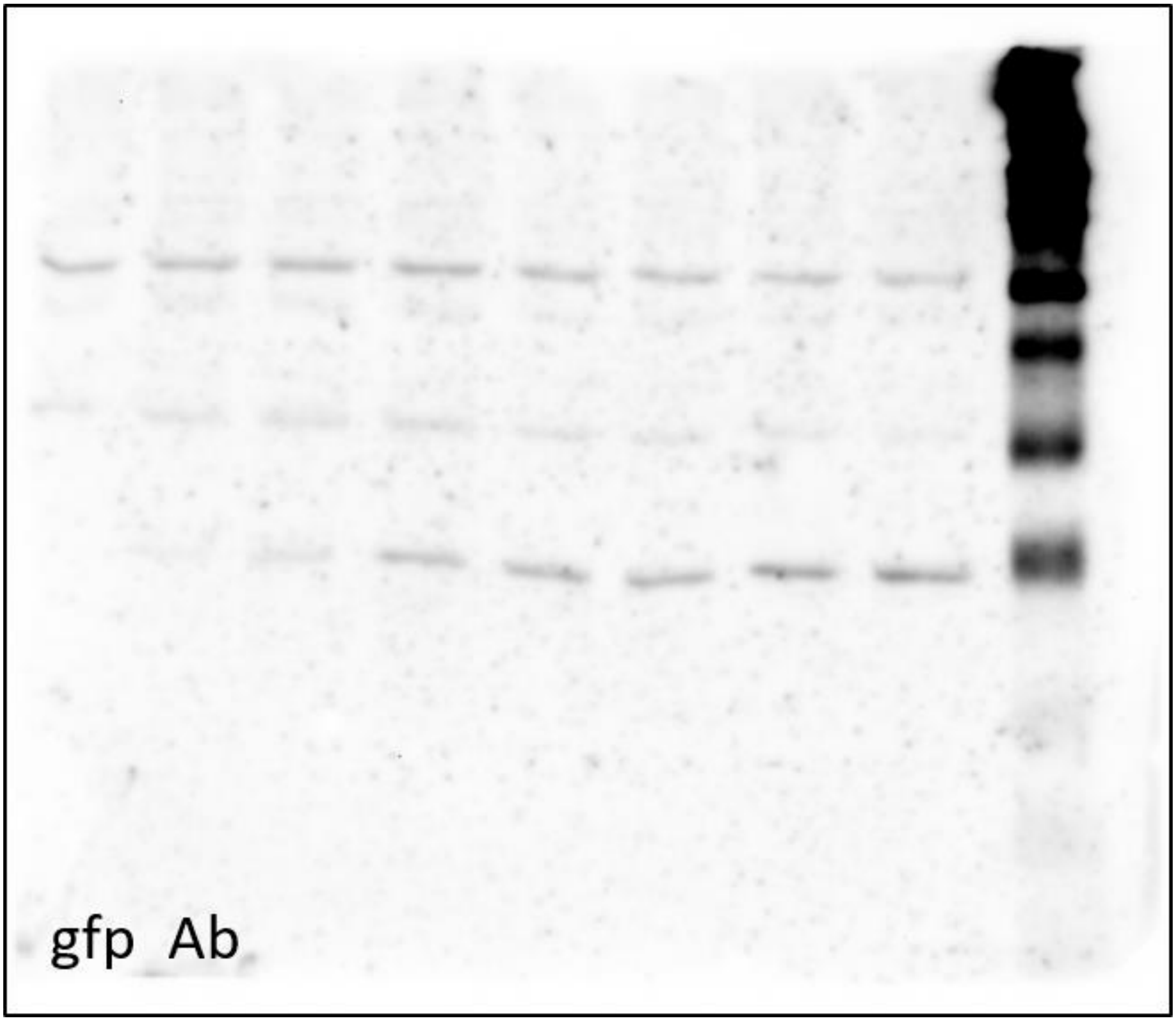

Suppl. material S9: Original blot used to generate Figure 6b

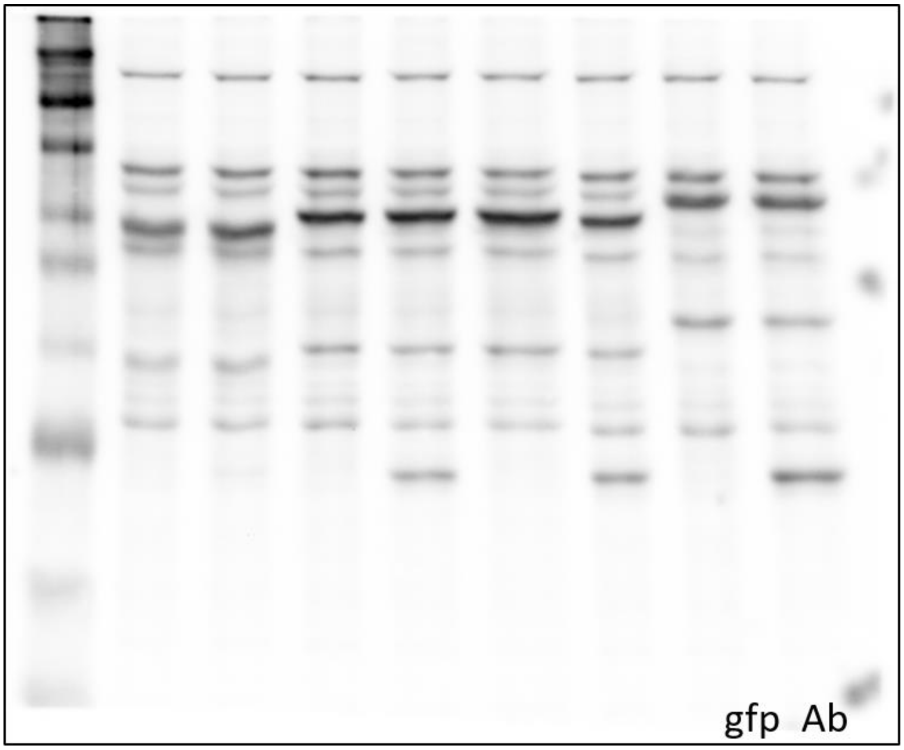

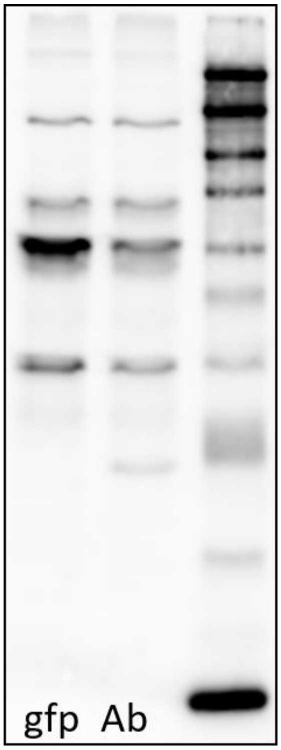

Suppl. material S10: Original blots used to generate Figure 7d
